# tDCS home based treatment following accelerated dTMS in the elderly depressed

**DOI:** 10.1192/j.eurpsy.2023.167

**Published:** 2023-07-19

**Authors:** C. Baeken

**Affiliations:** ^1^Department of Psychiatry UZGent, Ghent University, Ghent; ^2^Department of Psychiatry UZBrussel, Vrije Universiteit Brussel, Brussel, Belgium

## Abstract

**Abstract:**

With a growing number of elderly persons, geriatric depression - associated with important morbidity and mortality- is becoming a significant health problem. Given the risk of polypharmacy and increased side effects, alternative non pharmaceutical treatments such as repetitive transcranial magnetic stimulation (rTMS) and transcranial direct current stimulation (tDCS) may be solution. Recently, the FDA approved deep brain TMS (dTMS) for depression, not only stimulating deeper cortical areas but response and remission rates may be better, especially in elderly populations. Nevertheless, beneficial follow-up options following rTMS treatment remains to be determined. Therefore, one week after the last accelerated dTMS, all patients followed a 3 week open label tDCS with a home-use device. Study rationale and preliminary findings will be discussed.

**Disclosure of Interest:**

None Declared

